# Serious Bacterial Infections in Hospitalized Neonates in Eastern Ethiopia: Investigating the Emerging Pathogen *Pantoea dispersa* Compared With *Klebsiella pneumoniae*

**DOI:** 10.1111/tmi.70140

**Published:** 2026-04-21

**Authors:** Yunus Edris, Faisel A. Hassen, Desalegn A. Ayana, Fami Ahmed, Haleluya Leulseged, Dadi Marami, Jabir Aliye, Belete G. Alem, Zelalem T. Mariam, Gezahang Mengesha, Nega Assefa, Alexander M. Aiken, J. Anthony G. Scott, Lola Madrid

**Affiliations:** ^1^ London School of Hygiene and Tropical Medicine London UK; ^2^ Haramaya University College of Health and Medical Sciences Harar Ethiopia; ^3^ KEMRI‐Wellcome Trust Research Programme Kilifi Kenya

**Keywords:** bacteraemia, Ethiopia, infant mortality, *Klebsiella pneumoniae*, neonatal sepsis, *Pantoea dispersa*

## Abstract

**Background:**

Serious bacterial infections (SBIs) are major contributors to neonatal morbidity and mortality in low‐income countries. We describe the aetiology and risk factors for neonatal bacteraemia and in‐hospital mortality in eastern Ethiopia, focusing on *Pantoea dispersa,* a rarely studied pathogen, and *Klebsiella pneumoniae* .

**Methods:**

Prospective surveillance was conducted at Hiwot Fana Comprehensive Specialized Hospital (HFCSH), Harar, from December 2021 to November 2023. Blood for culture was drawn from neonates admitted with the WHO clinical definition of possible‐SBI (pSBI). Isolates were identified using API kits and antimicrobial susceptibility tested by Kirby‐Bauer method.

**Findings:**

Among 1375 neonates with pSBI, blood was cultured from 1335 (97%), and 356 (27%) cultured pathogens. The commonest infections were *Pantoea* species (*n* = 145, 40.7%) and *K. pneumoniae* (*n* = 63, 17.7%); 128 *Pantoea* isolates were identified as *P. dispersa* by Matrix‐Assisted Laser Desorption Ionization‐Time of Flight. Case‐fatality‐ratios were 25% (32/128), 19% (9/47) and 19% (30/160) for *P. dispersa* , *K. pneumoniae* and other monomicrobial infections, respectively. *P. dispersa* showed resistance to ampicillin (99%) and cefotaxime (85%) but was otherwise broadly susceptible, while *K. pneumoniae* showed resistance to cefotaxime (100%) and gentamicin (89%), remaining susceptible only to amikacin and meropenem. Compared to non‐bacteraemic admissions, *P. dispersa* bacteraemia was associated with health‐facility delivery outside HFCSH (aOR 1.9 [95% CI, 1.21–2.97]), low birth weight (aOR 2.1 [95% CI,1.22–3.47]), and the dry season (aOR 9.7, [95% CI, 4.61–20.31]). *K. pneumoniae* bacteraemia was associated with health‐facility delivery outside HFCSH (aOR, 2.2, [95% CI, 1.09–4.27]) alone. Among admissions with pSBI, death was associated with low birth weight and *P. dispersa* bacteraemia.

**Interpretation:**

Bacteraemia was prevalent among neonates admitted with pSBI to HFCSH. *P. dispersa* and *K. pneumoniae* predominated and both had high mortality risks*.* Rigorous diagnostics and epidemiological associations support the interpretation of *P. dispersa* as a pathogen, necessitating local investigation into transmission and infection control interventions.

## 1 Introduction

Approximately three million neonates develop serious bacterial infections (SBIs) globally each year, leading to at least 750,000 deaths, predominantly in low‐ and middle‐income countries (LMICs) [1]. In Ethiopia, limited access to diagnostics and effective antimicrobial therapies and scarcity of epidemiological data exacerbate SBI‐related neonatal mortality, impeding progress toward Sustainable Development Goal 3.2 [2]. The aetiology of neonatal SBIs varies by timing and region. Perinatal pathogens are derived mainly from maternal flora whilst postnatal pathogens are derived from hospital or community environments [3]. Gram‐positive bacteria predominate in high‐income countries (HICs) [4] whilst several multi‐centre studies, including ANISA, BARNARDS, and CHAMPS, have shown that Gram‐negative coliforms, particularly *K. pneumoniae* , predominate in LMICs [5, 6, 7, 8].

In Hiwot Fana Comprehensive Specialized Hospital (HFCSH), a referral hospital located in Eastern Ethiopia, we discovered a high burden of neonatal bacteraemia during the MBIRA study (Mortality from Bacterial Infections Resistant to Antibiotics), a multinational prospective cohort study on the impact of antimicrobial resistance on mortality [7]. In this hospital, in addition to 176 isolates of *K. pneumoniae* , there were 74 bacteraemia cases with *Pantoea species*, of which 71 were in neonates. Initially, we considered these *Pantoea* spp. as a contaminant as there were insufficient numbers to assess the case fatality ratio (CFR) and we excluded them from our analysis. However, their persistent isolation in 2022–2023 prompted further investigation into their clinical significance.

The genus *Pantoea* belongs to the order *Enterobacterales* and is frequently found in plants, soil and water. While primarily plant pathogens, at least seven of the 20 identified *Pantoea* spp. have been implicated in human infections, with *P. agglomerans, P. dispersa*, and *P. septica* most frequently isolated from clinical specimens [9]. *P. agglomerans* has been linked to neonatal nosocomial outbreaks, often via contaminated milk formula or parenteral nutrition, and is considered a rare opportunistic pathogen particularly in patients with underlying comorbidities [9, 10, 11]. *P. dispersa* , however, is less commonly reported but has caused bloodstream infections in neonates and adults, with genomic studies revealing virulence factors such as type VI secretion systems and iron uptake genes that increase pathogenicity in vulnerable hosts [12, 13, 14]. In general, well‐designed epidemiological studies on *Pantoea* spp. in LMICs are scarce, and no research has yet explored risk factors for *Pantoea* spp. bacteraemia or their impact on in‐hospital mortality against other well‐established neonatal pathogens, especially from sub‐Saharan Africa [9]. As *P. dispersa* infection has rarely been reported, its role in neonatal sepsis is poorly understood [13, 14]. Recognition of organisms as pathogens, as demonstrated with *Streptococcus pneumoniae*, [15] facilitates the design of targeted interventions, highlighting the need to clarify the potential causal role of *P. dispersa* in neonatal disease.

This study aimed to determine the aetiology of SBI in neonates admitted to HFCSH, focusing on the risk factors for *P. dispersa* bacteraemia and its impact on in‐hospital mortality, using *K. pneumoniae* as a well‐described comparator. The evidence from this study may help to inform clinical management and infection control strategies in similar LMICs settings.

## 2 Methods

### 2.1 Study Setting

The study was conducted at HFCSH in Harar, eastern Ethiopia, a city of 276,000 people. HFCSH has 371 adult beds and 129 paediatric beds, including a 40‐bed neonatal intensive care unit (NICU). It serves a catchment area of ~5.8 million people. The NICU receives ~750 admissions yearly and the commonest diagnoses are non‐exclusively SBI (~90%), prematurity (~37%), and birth asphyxia (~12%).

The NICU comprises seven rooms: three interconnected six‐bedded rooms for preterm, term and critical neonates, separated by aluminium partitions with open paths allowing movement of the care team between areas. The remaining four rooms are located on the same corridor, two housing four beds each and two for kangaroo mother care and stable neonates with mothers. Each room has functional handwashing sinks.

### 2.2 Study Design and Population

All neonates (aged 0–28 days) admitted to HFCSH between December 1, 2021, and November 30, 2023, were included in the surveillance if they met the World Health Organization's (WHO) criteria for possible serious bacterial infection (pSBI) [6, 16] and their family consented. pSBI is considered if the neonate has one or more of the following features not explained by another diagnosis: fast breathing, severe chest indrawing, no movement at all or movement only when stimulated, inability to feed at all or not feeding well, convulsions, fever (≥ 38°C) or hypothermia (< 35.5°C). In HFCSH, pSBI is treated with ampicillin and gentamicin as a first line as per WHO 2013 Guideline, [16] and adjusted according to the patient's clinical response and the culture results.

Data were collected on maternal sociodemographic status, labour and delivery, neonatal age, sex, gestational age, birth weight, presenting clinical conditions, and discharge outcome. Enrolled neonates were followed up throughout their stay in the hospital until discharge or death.

### 2.3 Blood Sample Collection and Laboratory Analysis

Blood samples were collected from eligible neonates within 48 h of admission to hospital. One to 3 mL of blood (minimum: 0.5 mL) for neonates was aseptically collected from a peripheral vein after disinfecting the venipuncture site with 70% alcohol followed by 2% chlorhexidine. Blood was inoculated into BACT/ALERT PF Plus (bioMérieux Inc., Durham, NC, USA) culture bottles and processed with the automated BACT/ALERT incubator for 5 days. Positive cultures underwent Gram‐staining and were sub‐cultured onto all available routine media (blood, chocolate, chromogenic, MacConkey and Xylose‐Lysine‐Deoxycholate (XLD) agar) to optimize pathogen recovery and incubated at 37°C in aerobic and CO2‐enriched conditions. Identification of suspected pathogens to species level was performed using Analytical Profile Index (API) biochemical identification kits (bioMérieux) as per the manufacturer's instructions. *Micrococcus* spp., *Bacillus* spp., *Diphtheroids*, *Corynebacterium* spp., coagulase‐negative *Staphylococcus* spp. (CoNS), *Propionibacterium*, [17] and unidentified Gram‐positive rods were considered as contaminants for all patients. We included *Candida* spp. identification in blood culture under the term “bacteraemia” and considered these to be pathogens.

Antimicrobial susceptibility testing (AST) was performed using the Kirby‐Bauer disc diffusion method with Oxoid diffusion discs (Thermo Fisher, Waltham, MA, USA) following Clinical and Laboratory Standards Institute guidelines [18]. The panel included ampicillin, co‐amoxiclav, ciprofloxacin, and cefotaxime for both groups; gentamicin, ceftazidime, amikacin, meropenem, and piperacillin–tazobactam for Gram‐negatives; and vancomycin, cotrimoxazole and cloxacillin for Gram‐positives. Additionally, *P. dispersa* and *K. pneumoniae* were tested for cefuroxime, ampicillin‐sulbactam, chloramphenicol, tetracycline, and doxycycline. The intermediate‐susceptibility status group was grouped with resistant isolates. We defined multidrug resistance (MDR) according to Magiorakos et al. [19], as non‐susceptibility to ≥ 1 agent in ≥ 3 antimicrobial categories.

The quality controls used were the appropriate American Type Culture Collection controls. Given the unexpectedly high frequency of *Pantoea* spp. isolation and concerns regarding possible misidentification, firstly, a random subset of 30 monomicrobial isolates was re‐identified using MicroScan WalkAway (Beckman Coulter, USA), and secondly, all monomicrobial *Pantoea* spp. isolates (*n* = 129) were analysed by Matrix‐Assisted Laser Desorption Ionization‐Time of Flight Mass Spectrometry (MALDI‐TOF MS), which we considered a superior identification method for this genus [14, 20]. The *Pantoea* spp. isolates identified in polymicrobial were not retested by MALDI‐TOF and hence were categorized as *Pantoea* spp. The polymicrobial cultures were not analysed using MALDI‐TOF MS because sub‐culturing does not fully address the technical limitations of MALDI‐TOF in polymicrobial contexts [21]. Additionally, our access to MALDI‐TOF was limited, prompting us to focus on isolates selected for downstream risk factor analyses.

The Hararghe Health Research Partnership laboratory, where most of this work was conducted, was established in 2017 as part of a research collaboration between London School of Hygiene and Tropical Medicine (LSHTM), UK and Haramaya University, Ethiopia. This laboratory is accredited to ISO 15189 and undergoes regular external quality control assessment by the UK National External Quality Assessment Service (UK‐NEQAS). MALDI‐TOF analysis was performed externally at the Animal Health Institute (AHI), in Addis Abba, Ethiopia. This laboratory, established in 2021, integrates advanced diagnostic techniques and innovative research methodologies to enhance livestock productivity and disease management (https://www.ahi.gov.et/).

### 2.4 Statistical Analysis

All analyses were performed using STATA SE version 18. We used descriptive statistics to summarize the demographic, clinical, and identified pathogens. We then undertook two multivariable analyses: (i) risk factors for admission with *P. dispersa* or *K. pneumoniae* bacteraemia; (ii) risk factors for death following bacteraemia with either *P. dispersa* or *K. pneumoniae* . Other causes of bacteraemia were excluded from these analyses as their role in clinical outcomes in neonates is well‐described elsewhere [6, 8]. Polymicrobial bacteraemia were also excluded from these analytic models for simplicity and because they were relatively infrequent.

Multinomial logistic regression was used to assess the risk factors for bacteraemia on admission due to either *P. dispersa* or *K. pneumoniae* compared against non‐bacteraemic admissions with pSBI. Model outputs are presented as odds ratios (ORs) with 95% confidence intervals (CIs). The impacts of *P. dispersa* and *K. pneumoniae* bacteraemia on inpatient mortality were evaluated using binomial regression with a log‐link. The analysis compared the mortality of children admitted with pSBI and monomicrobial cultures of *P. dispersa* or *K. pneumoniae* against children admitted with pSBI and negative blood cultures. Model outputs are presented as risk ratios (RRs) with 95% CIs. In both models, variables with a *p* < 0.1 in the univariate analysis were included in the multivariable models.

Missing values were coded separately for the place of delivery, birth weight, and gestational age. Season was categorized into three strata: October to January, February to May, and June to September, characterized by no rain, light rains, and heavy rains respectively [22].

This study was approved by the Research Ethics Review Committee at Haramaya University's College of Health and Medical Sciences and the LSHTM (Reference 14,394–3). For each child investigated, a parent/guardian provided written informed consent.

### 2.5 Funding Source

This work was supported by the Gates Foundation [Grant reference numbers OPP1126780 and 100,548]. JAGS is funded as a fellow by the Wellcome Trust [214320]. The funding organizations provided input on study design but had no role in data collection, analysis, or interpretation.

## 3 Results

**FIGURE 1:**
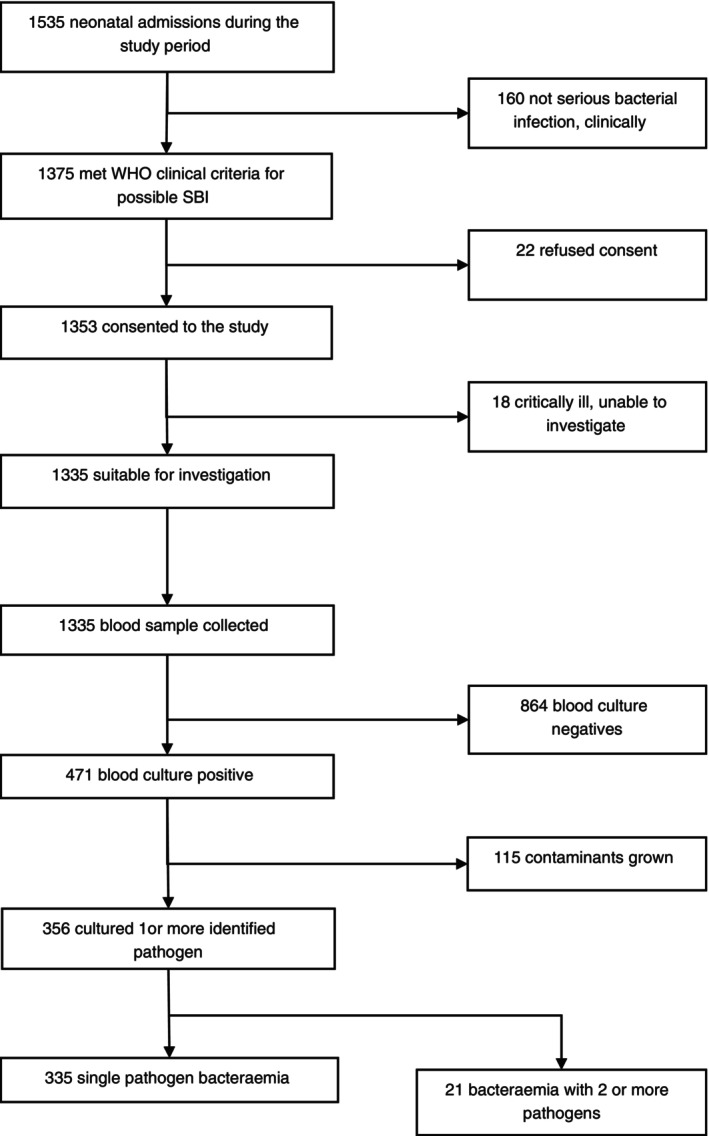
Flow diagram of study participants.

From December 1, 2021, to November 30, 2023, a total of 1535 neonates were hospitalized in HFCSH; 1375 (90%) were diagnosed with pSBI, and blood culture was collected in 1335 (87%, Figure 1); consent was declined by parents of 22 neonates, and a blood sample could not be obtained from 18 because of the severity of their illness. Among 1335 blood cultures taken, 356 (27%) grew at least one pathogen, including 21 samples with polymicrobial infection, and 115 (8.6%) grew contaminants; these were excluded from later analysis, giving a bacteraemia proportion of 29% (356/1220) (Figure 1).

Among 1220 neonates analysed, 517 (42.4%) were female, 436 (35.7%) had low birth weight, 491 (40.2%) were born prematurely, 719 (58.9%) were born in HFCSH, and 398 (32.6%) were admitted during Ethiopia's heavy rainy season (June–September, Table 1).

**TABLE 1:** Baseline characteristics of 1220 neonates stratified by the blood culture results.

Characteristics	Bloodstream infection, *n* (%)					
	Total (*n* = 1220)	Non‐bacteraemic (*n* = 864)	*P. dispersa* (*n* = 128)	*K. pneumoniae* (*n* = 47)	Other pathogens (*n* = 160)	Polymicrobial (*n* = 21)
Place of delivery						
Hiwot Fana Hospital	719 (58.9%)	529 (61.2%)	67 (52.3%)	21 (44.7%)	90 (56.2%)	12 (57.1%)
Home	93 (7.6%)	65 (7.5%)	6 (4.7%)	3 (6.4%)	17 (10.6%)	2 (9.5%)
Other facilities	400 (32.8%)	265 (30.7%)	53 (41.4%)	23 (48.9%)	52 (32.5%)	7 (33.3%)
Unknown	8 (0.7%)	5 (0.6%)	2 (1.6%)	0 (0.0%)	1 (0.6%)	0 (0.0%)
Neonatal characteristics						
Age in days						
0–3	903 (74.0%)	639 (74.0%)	101 (78.9%)	33 (70.2%)	117 (73.1%)	13 (61.9%)
4–28	317 (26.0%)	225 (26.0%)	27 (21.1%)	14 (29.8%)	43 (26.9%)	8 (38.1%)
Sex						
Female	517 (42.4%)	352 (40.7%)	60 (46.9%)	21 (44.7%)	73 (45.6%)	11 (52.4%)
Season						
Oct–Jan (dry)	360 (29.5%)	233 (27.0%)	61 (47.7%)	16 (34.0%)	43 (26.9%)	7 (33.3%)
Feb–May (mild rainy)	462 (37.9%)	313 (36.2%)	58 (45.3%)	17 (36.2%)	62 (38.8%)	12 (57.1%)
Jun–Sep (heavy rainy)	398 (32.6%)	318 (36.8%)	9 (7.0%)	14 (29.8%)	55 (34.4%)	2 (9.5%)
Gestational age						
< 37	491 (40.2%)	329 (38.1%)	65 (50.8%)	19 (40.4%)	69 (43.1%)	9 (42.9%)
> 37	651 (53.4%)	479 (55.4%)	53 (41.4%)	22 (46.8%)	86 (53.8%)	11 (52.4%)
Unknown	78 (6.4%)	56 (6.5%)	10 (7.8%)	6 (12.8%)	5 (3.1%)	1 (4.8%)
Birth weight						
< 2500 g	436 (35.7%)	289 (33.4%)	63 (49.2%)	16 (34.0%)	55 (34.4%)	13 (61.9%)
> 2500 g	520 (42.6%)	393 (45.5%)	42 (32.8%)	18 (38.3%)	62 (38.8%)	5 (23.8%)
Unknown	264 (21.6%)	182 (21.1%)	23 (18.0%)	13 (27.7%)	43 (26.9%)	3 (14.3%)
Outcome						
Died	179 (14.7%)	99 (11.5%)	32 (25.0%)	9 (19.1%)	30 (18.8%)	9 (42.9%)

### 3.1 Type of Bloodstream Infection on Admission and Associated Risk Factors

Among 356 neonates with bacteraemia, the most commonly isolated pathogens were *Pantoea* spp. (*n* = 145, 40.4%) and *K. pneumoniae* (*n* = 63, 17.7%). Among 30 randomly selected monomicrobial *Pantoea* spp. isolates from blood cultures re‐identified by MicroScan, all were classified as *P. agglomerans* . However, MALDI‐TOF MS identified 128 of 129 monomicrobial *Pantoea* spp. isolates, including the same 30 isolates that were analysed by MicroScan, as *P. dispersa* , with one yielding a negative result. Other pathogens isolated are detailed in Table 2. The number of bacteria identified monthly during the study period can be found, by major species group, in Figure S1. *Candida* species were also frequent (*n* = 95, 26.7%) while Gram‐positive pathogens were rare, the commonest being *Staphylococcus aureus* (*n* = 5, 1.4%).

**TABLE 2:** Pathogens isolated in 356 neonates with pathogenic bacteraemia.

Pathogens identified	Total (*n*)	Deaths (*n*)	CFR (%)^d^	95 CI^e^
Monomicrobial infections	335	71	21.2	17–26
Gram‐negative	225	55	24.4	19–31
*Pantoea dispersa*	128	32	25.0	18–33
*Klebsiella pneumoniae*	47	9	19.1	8–30
*Klebsiella oxytoca*	9	3	33.3	3–64
*Serratia* species	11	2	18.2	0–41
*Escherichia coli*	9	3	33.3	3–64
Other Gram negatives^a^	21	6	28.6	9–48
Gram positive	16	3	18.8	4–46
*Staphylococcus aureus*	5	1	20.0	0–55
Other Gram positives^b^	11	2	18.2	0–41
Fungal infections	94	13	13.8	7–21
*Candida* species	94	13	13.8	7–21
Polymicrobial infections	21	9	42.9	22–66
*Klebsiella pneumoniae* and *Pantoea* species	15	6	40.0	15–65
Other polymicrobial^c^	6	3	50.0	10–90

^a^ This group includes *Enterobacter cloacae* (9), *Burkholderia cepacia (5)*, *Acinetobacter baumannii* (3), *Salmonella enteritica* (1), *Rahnella aquatilis* (2), and *Neisseria meningitidis* (1).

^b^ This group includes Group A *Streptococcus* (2), Group B *Streptococcus* (2), *Enterococcus* species (5), and *Streptococcus pneumoniae* (2).

^c^ This group includes *Pantoea* species+ *Acinetobacter baumannii* (2), *Candida* spp.+ *Kocuria varians* (1), *Serratia rubidaea* +*Salmonella enteritica* (1), *Klebsiella pneumoniae* + *Acinetobacter baumannii* (1), and *Enterococcus* species+ *Enterobacter cloacae* (1).

^d^ CFR: case fatality ratio.

^e^ CI: confidence interval.

We examined risk factors for monomicrobial infection with confirmed *P. dispersa* (*n* = 128) or *K. pneumoniae* (*n* = 47) against non‐bacteraemic pSBI patients (*n* = 864; total for multivariable analysis *n* = 1039). Multivariable analysis demonstrated that birth weight < 2500 g (aOR 2.1 [95% CI, 1.22–3.47]) and delivery at a health facility other than HFCSH (aOR 1.9, [95% CI, 1.21–2.97]) were associated with a *P. dispersa* bacteraemia. Additionally, admission during the no rain season (October–January, aOR 9.7; 95% CI, 4.6–20.3) or the light rains season (February–May, aOR 7.1, [95% CI, 3.4–14.6]) were associated with a *P. dispersa* bacteraemia compared to the heavy rainy season (June–September), was independently associated with increased odds of *P. dispersa* bacteraemia. By comparison, *K. pneumoniae* bacteraemia was only associated with delivery at non‐HFCSH health facilities (aOR 2.2, [95% CI, 1.09–4.27], Table 3).

**TABLE 3:** Univariate and multivariate analysis of factors associated with bloodstream infection on admission (*n* = 1039).

Characteristic	*K. pneumoniae* (= 47)					*P. dispersa* (*n* = 128)				
	Odds ratio		Adjusted odds ratio			Odds ratio		Adjusted odds ratio		
	OR (95 CI)	p	aOR	(95 CI)	p	aOR (95 CI)	p	aOR	(95 CI)	p
Age										
0–3 days	0.8 (0.44–1.5)	0.57				1.3 (0.84–2.0)	0.23			
Sex										
male	0.9 (0.47–1.54)	0.59				0.8 (0.54–1.13)	0.19			
Season										
Feb–May (light rains)	0.8 (0.39–1.60)	0.51	0.8	(0.40–1.67)	0.58	0.7 (0.48–1.05)	0.09	0.7	(0.48–1.09)	0.13
June‐Sept (heavy rains)	0.6 (0.31–1.34)	0.24	0.7	(0.31–1.41)	0.28	0.1 (0.05–0.22)	< 0.0001	0.1	(0.05–0.22)	< 0.0001
Gestational age										
Preterm (< 37 weeks)	1.3 (0.67–2.36)	0.48	1.4	(0.66–3.06)	0.37	1.8 (1.21–2.63)	< 0.0001	1.4	(0.84–2.28)	0.21
Unknown	2.3 (0.91–6.00)	0.08	2.2	(0.84–5.80)	0.11	1.6 (0.78–3.35)	0.20	1.2	(0.55–2.59)	0.65
Place of delivery										
Home	1.2 (0.34–4.01)	0.81	1.1	(0.29–4.09)	0.90	0.7 (0.30–1.75)	0.48	1.0	(0.37–2.44)	0.92
Other facilities	2.2 (1.19–4.02)	0.01	2.2	(1.09–4.27)	0.03	1.6 (1.07–2.33)	0.02	1.9	(1.21–2.97)	< 0.0001
Unknown	0.0	0.98	0.0	(0.00‐.)	0.98	3.2 (0.60–16.60)	0.17	7.5	(1.17–47.58)	0.03
Birth weight										
< 2500 g	1.2 (0.61–2.41)	0.59	1.1	(0.49–2.53)	0.80	2.0 (1.34–3.10)	< 0.0001	2.1	(1.22–3.47)	< 0.0001
Unknown	1.6 (0.75–3.25)	0.24	1.2	(0.54–2.76)	0.63	1.2 (0.69–2.02)	0.54	1.2	(0.66–2.19)	0.54

*Note:* Both the unadjusted and adjusted models were analysed using a multinomial logistic regression model. The reference groups were age‐4–28 days, sex‐female, season‐Oct–Jan (dry season) or no rains, birth weight‐ ≥ 2500 g, gestational age‐ > 37 weeks, rupture of membranes‐ < 12 h, place of delivery‐Hiwot Fana Hospital, and blood culture results—non‐bacteraemic. Adjusted for season of the year, gestational age, place of delivery, and birth weight.

Abbreviations: aOR, adjusted odds ratio; CI, confidence interval.

### 3.2 Inpatient Mortality and Associated Risk Factors

Among the 1220 neonates with pSBI, 179 died in the hospital (14.7%, Table 1). Among 356 neonates with bacteraemia on admission, 80 died (22.5%). The highest CFR was observed in patients with polymicrobial bacteraemia (42.9%, 9/21), followed by Gram‐negative bacteria (24.0%, 55/225). The lowest CFR was among those with negative blood cultures (11.5%, 98/864). Among the common Gram‐negative bacteria, *P. dispersa* had the highest CFR at 25.0% (32/128), followed by *K. pneumoniae* at 19.1% (9/47, Table 2).

We examined risk factors for mortality among neonates with clinically defined pSBI and with either negative blood cultures (*n* = 864) or with single‐pathogen bacteraemia caused by *K. pneumoniae* (*n* = 47) or *P. dispersa* (*n* = 128). In this multivariable model, low birth weight (aRR 1.9, 95% CI 1.19–3.02) and *P. dispersa* bacteraemia (aRR 1.77, 95% CI 1.20–2.61) were associated with mortality. The adjusted mortality risk ratio for *K. pneumoniae* bacteraemia was 1.6 (95% CI 0.87–3.01, Table 4).

**TABLE 4:** Univariate and multivariate analysis of factors associated with in‐hospital mortality (*n* = 1039).

Characteristics	Risk ratio			Adjusted risk ratio		
	RR	95 CI	*p*	aRR	95 CI	*p*
Age						
0–3 days	1.6	1.05–2.38	0.03	1.4	0.85–2.28	0.19
Sex						
Male	1.3	0.90–1.72	0.18			
Season						
Jun–Sep (rainy)	0.7	0.47–1.02	0.07	1.3	0.83–1.97	0.27
Feb–May (mild rain)	0.8	0.58–1.19	0.31	1.1	0.73–1.67	0.64
Birth weight						
< 2500	2.3	1.63–3.36	< 0.0001	1.9	1.19–3.02	0.01
Unknown	1.4	0.90–2.29	0.12	1.7	1.03–2.81	0.04
Gestational ages						
Preterm (< 37 weeks)	1.8	1.34–2.54	< 0.0001	1.2	0.74–1.90	0.48
Unknown	1.0	0.46–2.03	0.92	0.8	0.39–1.72	0.60
Place of delivery						
Home	0.9	0.49–1.77	0.82			
Other facilities	1.1	0.79–1.52	0.59			
Unknown	1.1	0.18–6.76	0.93			
Blood culture results						
*P. dispersa*	2.2	1.53–3.11	< 0.0001	1.8	1.20–2.61	< 0.0001
*K. pneumoniae*	1.7	0.90–3.10	0.10	1.6	0.87–3.01	0.13

*Note:* The Unadjusted and Adjusted models were analysed using a binomial family with a log link regression model. The reference groups were age‐4–28 days, sex‐female, season‐Oct–Jan (dry season) or no rains, birth weight‐ ≥ 2500 g, gestational age‐ > 37 weeks, place of delivery‐Hiwot Fana Hospital and blood culture results—non‐bacteraemic. Adjusted for age group, season of the year, birth weight, gestational age, and blood culture results.

Abbreviations: aRR, adjusted risk ratio; CI, confidence interval.

### 3.3 Antibiotic Susceptibility Patterns of *P. dispersa* and *K. pneumoniae*

The antimicrobial resistance patterns of *P. dispersa* and *K. pneumoniae* exhibited distinct phenotypes (Table 5). Of 96 isolates of *P. dispersa* , 95 (99%) were resistant to ampicillin and 85 (88.5%) were resistant to cefotaxime. These isolates were largely susceptible to tetracycline, gentamicin, amikacin, ciprofloxacin, and meropenem, resulting in a low MDR phenotype prevalence of 5.8% (4/69) (Table 6).

**TABLE 5:** Antimicrobial susceptibility profiles of the *P. dispersa* and *K. pneumoniae* isolates.

Antimicrobial category	Antimicrobial agent	*P. dispersa*	*K. pneumoniae*
		Resistant *n*/*N* (%)	Resistant *n*/*N* (%)
Penicillin	Ampicillin	95/96 (98.9%)	28/28 (100.0%)
Penicillin + β‐lactamase inhibitors	Ampicillin sulbactam	49/96 (51.0%)	43/43 (100.0%)
	Amoxicillin‐clavulanic acid	51/96 (53.1%)	24/27 (88.9%)
Aminoglycosides	Gentamicin	2/95 (2.1%)	25/28 (89.3%)
	Amikacin	2/93 (2.2%)	3/28 (10.7%)
Fluoroquinolones	Ciprofloxacin	3/95 (3.2%)	11/28 (39.3%)
Cephalosporins	Cefuroxime	96/96 (100.0%)	43/43 (100.0%)
	Cefotaxime	85/96 (88.5%)	43/43 (100.0%)
	Ceftazidime	5/95 (5.3%)	27/28 (96.4%)
Carbapenems	Meropenem	1/93 (1.1%)	1/29 (3.4%)
Phenicols	Chloramphenicol	2/96 (2.1%)	19/43 (44.2%)
Tetracyclines	Doxycycline	0/96 (0.0%)	31/43 (72.1%)
	Tetracycline	0/96 (0.0%)	30/43 (69.8%)

*Note: n* = number of resistant isolates; *N* = total number of isolates tested for the specific antimicrobial agent. The number of respective tested antibiotics does not match the total isolates for *P. dispersa* (*n* = 128) and *K. pneumoniae* (*n* = 47), as there was a missing number for the tested antibiotics. This variation of the *N* denominator was due to interruptions in the supply of susceptibility testing discs during the course of the project.

**TABLE 6:** Distribution of isolates by number of antimicrobial classes with observed resistance.

Pathogen	Classes of antimicrobials agents						
	2 classes *n* (%)	3 classes *n* (%)	4 classes *n* (%)	5 classes *n* (%)	6 classes *n* (%)	7 classes *n* (%)	MDR overall *n* (%)
*P. dispersa* (*n* = 69)	65 (94.2)	3 (4.4)	1 (1.5)	0 (0.0)	0 (0.0)	0 (0.0)	4 (5.8)
*K. pneumoniae* (*n* = 25)	1 (4.0)	6 (24.0)	3 (12.0)	9 (36.0)	5 (20.0)	1 (4.0)	24 (96.0)

*Note:* 2, 3…,7 classes stand for resistance to 2,3…, 7 antibiotics from different classes. Denominators (*n*) represent the subset of isolates with complete susceptibility data across all tested antimicrobial categories (69/128 for *P. dispersa* and 25/47 for *K. pneumoniae* ). For *K. pneumoniae* , MDR profiling was performed across 7 categories (excluding ampicillin due to *K. pneumoniae* 's near total resistance to it). For *P. dispersa* , 8 categories were evaluated.

Conversely, *K. pneumoniae* isolates showed near‐complete resistance to cefotaxime (25/25, 100%) and gentamicin (25/28, 89.3%). Unlike *P. dispersa, K. pneumoniae* showed high resistance to tetracyclines (69.8%) and chloramphenicol (44.2%). Amikacin (89.3% susceptible) and meropenem (96.6% susceptible) were the only agents that remained effective. Consequently, 96.0% (24/25) of *K. pneumoniae* isolates were classified as MDR, mainly involving co‐resistance to beta‐lactams and aminoglycosides.

## 4 Discussion

This study of pSBI in neonates provides a detailed picture of aetiology at a large referral hospital in eastern Ethiopia, where previous clinical and microbiological data are sparse. It confirms, as seen elsewhere [1] that SBI is a significant cause of neonatal death. The pattern of pathogens is similar to that published in other studies in Africa [8] with the notable exception that *P. dispersa* emerged as the commonest cause of bacteraemic and fatal pSBI. Low birth weight was a risk factor for *P. dispersa* bacteraemia and delivery outside HFCSH was a risk factor for both *P. dispersa* and *K. pneumoniae* bacteraemia. Low birth weight and *P. dispersa* bacteraemia were independently linked to in‐hospital mortality.

Although *P. dispersa* bacteraemia has been reported only rarely in the past, [13, 14] we believe that it is behaving as a true pathogen, rather than a contaminant or incidental finding, in our neonatal patients; this conclusion is supported by robust laboratory, clinical and epidemiological evidence. Although performance comparisons for laboratory methods for identification of *Pantoea* spp. are limited, the concordance between the results of MALDI‐TOF and whole genome sequencing is 76% (40/67) when *P. agglomerans* is dominant, [9] and 90% (19/21) when *P. dispersa* is prevalent [14]. Our consistent genus‐level identification between API, Microscan, and MALDI‐TOF MS, with over 99% concordance, mitigates concerns of misidentification commonly associated with culture‐based methods [9]. Clinically, the high CFR of 25.0% (32/128) for *P. dispersa* bacteraemia, exceeding *K. pneumoniae* bacteraemia (19.1%) and non‐bacteraemic pSBI (11.5%) strongly implies pathogenic behaviour and suggests clinical or laboratory contamination is an unlikely explanation. Furthermore, the epidemiological parallels with *K. pneumoniae* —including shared associations with low birth weight and nosocomial acquisition—also support its role as a bacterial pathogen of neonates [23, 24]. Genomic analysis elsewhere, including work on bacteraemia isolates collected in other Ethiopian hospitals, indicates that *P. dispersa* can possess genes related to antibiotic resistance and virulence, consistent with its potential to cause serious infections [12, 14, 25]. Reports of *P. dispersa* causing neonatal sepsis in India and Yemen further support its international clinical relevance [13, 26]. Additionally, we conducted a similar study involving older children in the same setting, which identified six cases of *Pantoea* species, reinforcing a potential preference of *P. dispersa* for vulnerable neonates [27].

The high proportion of neonatal admissions with bacteraemia (29%) exceeds reports from similar settings in LMICs (9%–25%) [24, 28]. The unprecedented prevalence of *P. dispersa* suggests unusual local circumstances, potentially reflecting some unique environmental factors, such as the prolonged 2020–2023 drought in eastern Ethiopia [22] or healthcare‐related issues like inadequate infection control, [29] underscoring the need for region‐specific surveillance to inform targeted interventions. The *P. dispersa* isolates we identified were susceptible to most of the tested antibiotics other than ampicillin and cefotaxime whereas *K. pneumoniae* exhibited resistance to most agents, with only meropenem and amikacin consistently found as suitable treatment options.

Regarding bacterial transmission, delivery in another facility was associated with an increased risk of *K. pneumoniae* or *P. dispersa* bacteraemia. This suggests that nosocomial transmission of these organisms may be occurring outside of the HFCSH. Nosocomial transmission of pathogens causing bacteraemia in neonates is well‐recognized in LMIC [17, 23]. Specifically, outbreaks of *P. agglomerans* have been reported related to contamination of intravenous fluid, milk formula, or parenteral nutrition [9]. In this series, there were insufficient clinical records from the referring health centres to investigate these hypotheses retrospectively. The epidemiological association with low birthweight mirrors the risk profiles of established nosocomial pathogens, like *K. pneumoniae* , and opportunistic pathogens, like *P. agglomerans* [9, 23].

In this study, admission between October and January (dry) and between February and May (light rains) were both strongly associated with hospitalization due to *P. dispersa* bacteraemia in comparison to the June–September period (heavy rains). The higher risk of *P. dispersa* bacteraemia correlation with season may reflect reduced water availability, which limits hygiene practices and facilitates bacterial transmission, aligning with longstanding evidence that water access and sanitation are key to infection control [30]. Environmental factors such as dust and temperature variation may also play a role, as reported for related species like *P. agglomerans* [31].

The study results show that *P. dispersa* and low birth weight were independently associated with increased risk of in‐hospital death. This is consistent with prior studies where low birth weight [24] and bacteraemia due to Enterobacterales—the bacterial order that includes *P. dispersa* —were linked with an increased risk of in‐hospital death [7]. Specifically, while *P. dispersa* ‐bacteraemia related neonatal deaths have been reported elsewhere in Ethiopia, [14] and in other countries, a review of 10 published case reports identified three deaths, though most of these case were in adults [13]. The high CFR of *P. dispersa* , despite its susceptibility to locally available antimicrobials, suggests that mortality is influenced by patient vulnerability and health system factors rather than by antimicrobial resistance alone. This is consistent with findings from the MBIRA study, which showed no significant mortality differences between bacteraemia due to third‐generation cephalosporins resistant and susceptible Enterobacterales [7]. Unlike *K. pneumoniae* , which was associated only with outborn delivery, *P. dispersa* bacteraemia was linked to both low birth weight and outborn status—two known factors associated with poorer outcomes [24, 32]. These findings emphasize the need to strengthen health systems holistically to ensure timely diagnosis, referral, and supportive care. The CHAMPS network similarly reported that 77% of deaths among children under five and stillbirths were potentially preventable, with 75% related to stillbirths and neonatal deaths—emphasizing the importance of improving antenatal care, health education, and case management to reduce preventable death [33].

To our knowledge, this is the largest prospective series of *P. dispersa* bacteraemia ever reported. By using three different methods of identification across two laboratories we have identified these isolates with reasonable certainty. Previous studies, conducted in HIC or LMICs, have typically described small case series of bacteraemia with *P. dispersa* in neonates [26] identifying a low prevalence of this organism [13].

Our study has some limitations as follows. First, blood cultures are insensitive, which can lead to misclassification of some bacteraemic patients. Unavoidable challenges including low blood volume collection from small neonates and prior antibiotic use exacerbate this issue. As a result, some true cases of bacteraemia may go undetected, leading to an underestimation of the burden of bacteraemia. Some culture‐negative patients may therefore have bacteraemia, potentially biasing the risk factor analyses toward the null. Second, due to a contamination rate of 8.6% (115/1335), which is similar to or lower than other studies in comparable LMICs settings (e.g., 9.0%, 16.2%), [34, 35] some patients were excluded from some analyses. Moreover, we did not perform genomic sequencing of our isolates, which could have elucidated virulence mechanisms; however, recent genomic studies on similar Ethiopian *P. dispersa* isolates support their pathogenic potential [14]. We included nearly 90% of all neonatal admissions with pSBI in our main analysis dataset, which forms a robust basis for evaluating the risks associated with *P. dispersa* bacteraemia and in‐hospital death.

In summary, this study identifies *P. dispersa* as a significant contributor to neonatal bacteraemia and mortality in eastern Ethiopia. The clinical behaviour and epidemiology of *P. dispersa* appear similar to those of *K. pneumoniae* , currently considered the largest infectious killer of neonates in LMICs, with a tropism for vulnerable neonates, an association with prior health‐care contact and a high CFR, underscoring the need for enhanced surveillance and infection prevention measures. Future research should explore the incidence, transmission, and virulence mechanisms of *P. dispersa* in this region and elsewhere to better understand its pathogenicity and set strategies for prevention.

## Funding

This work was supported by Bill and Melinda Gates Foundation, OPP1126780.

## Conflicts of Interest

The authors declare no conflicts of interest.

## Supporting information


**Figure S1:** Number of isolates of monomicrobial *P. dispersa* compared to *K. pneumoniae* and other pathogenic bacteria by month from December 2021 to November 2023 in the NICU of HFCSH.

## Data Availability

The supporting information to help the reader are publicly available online. The corresponding author should address any questions or comments. The full data set for research or evaluation purposes can be accessed from the Hararghe Health Research Partnership (HHR) after contacting the corresponding author.
