# Supplementary material for: Serious Bacterial Infections in Hospitalized Neonates in Eastern Ethiopia: Investigating the Emerging Pathogen Pantoea dispersa Compared With Klebsiella pneumoniae
Source: Trop Med Int Health. 2026 Apr 21;31(7):933–43. doi: 10.1111/tmi.70140 (PMC13331537; doi:10.1111/tmi.70140)
Supplement: Supplementary file 1 — Figure S1: Number of isolates of monomicrobial P. dispersa compared to K. pneumoniae and other pathogenic bacteria by month from December 2021 to November 2023 in the NICU of HFCSH. [file TMI-31-933-s001.docx]

**Supplementary Information**

**Serious bacterial infections in hospitalised neonates in eastern Ethiopia: investigating the emerging pathogen *Pantoea dispersa* compared with *Klebsiella pneumoniae***

**Authors:** Yunus Edris, MD^1,2^, Faisel A. Hassen, MD^2^, Desalegn A. Ayana, PhD^2^, Fami Ahmed, BSc^2^, Haleluya Leulseged, MD^1,2^, Dadi Marami, MSc^2^, Jabir Aliye, MD^1^, Belete G. Alem, MD^2^, Zelalem T. Mariam, MSc^2^, Gezahang Mengesha, MSc^2^, Nega Assefa, PhD^1,2^, Alexander M. Aiken, PhD^2^, J. Anthony G. Scott, FMedSci^†1,3^, Lola Madrid, PhD^†1^

**Authors Affiliations**:

^1^London School of Hygiene & Tropical Medicine, London, UK.

^2^Haramaya University, College of Health and Medical Sciences, Harar, Ethiopia.

^3^KEMRI-Wellcome Trust Research Programme, Kilifi, Kenya.

**Corresponding author:**

* Yunus Edris Kelil

**Email:** [Yunus-Edris.Kelil@lshtm.ac.uk](mailto:Yunus-Edris.Kelil@lshtm.ac.uk)

**Phone:** +251945593004

ORCiD: <https://orcid.org/0000-0001-9455-8109>

^†^J. Anthony G. Scott and Lola Madrid are joint senior authors

Contents

[Supplementary Figure S1: Number of isolates of monomicrobial *P. dispersa* compared to *K. pneumoniae* and other pathogenic bacteria by month from December 2021 to November 2023 in the NICU of HFCSH. 2](#_Toc222892713)

# **Supplementary Figure S1: Number of isolates of monomicrobial *P. dispersa* compared to *K. pneumoniae* and other pathogenic bacteria by month from December 2021 to November 2023 in the NICU of HFCSH.**
